# Is there any job for me? Qualitative exploration of support needs among young Swedish adults with psychosis envisioning productive activities

**DOI:** 10.3233/WOR-230311

**Published:** 2024-07-03

**Authors:** A. Birgitta Gunnarsson, Jan-Åke Jansson, Mona Eklund

**Affiliations:** a Department of Research and Development, Region Kronoberg, Växjö, Sweden; b Department of Clinical Neuroscience and Rehabilitation, University of Gothenburg, Gothenburg, Sweden.; c Department of Psychology, Lund University, Lund, Sweden; d Department of Health Sciences, Lund University, Lund, Sweden

**Keywords:** Activity, employment, interviews, mental health, occupations, qualitative research, work

## Abstract

**BACKGROUND:**

Unemployment is high not only among people with mental illness, but also among young adults in general. The combination of having a severe mental illness and being young entails a particularly problematic situation for young people with psychosis. This study aimed to understand how this group envision their future possibilities for entering the labour market or engaging in other productive activities.

**OBJECTIVE:**

To explore how young adults with psychosis perceive their possibilities, wishes and support needs for gaining employment or engaging in other productive activities.

**METHODS:**

A descriptive design with qualitative individual in-depth interviews was used. Eighteen young adults with psychosis, aged 18–30 years, were interviewed. Data was analysed with qualitative content analysis.

**RESULTS:**

Four categories based on the experiences of the participants as being vital for having work or other productive activities were generated: “Wishing for a role in the community”, “Being my own coach”, “Needing personal support” and “Having a supportive workplace”. Each of them included sub-categories.

**CONCLUSION:**

The young adults with psychosis were a long way from having paid work, but they desired to have a worker role in the community. They are a heterogeneous group, which entails that it is important that professionals and employers have a person-centered and holistic approach, listening to the individuals themselves.

## 1 Background

Young adults with psychosis or other severe and persistent mental illnesses are at risk of a psychiatric disability, defined by the Swedish Social Board of Health and Welfare as a condition that lasts for considerable time and causes restrictions in coping with everyday life [1]. An important consequence is the threat that they will never complete their education and thus have difficulty in entering the labour market [2, 3]. Unemployment is high not only among people with mental health issues, but also among young adults in general, and the combination of having a severe mental illness and being young entails a problematic situation [3, 4]. Having employment is essential for human beings [5]. Salaried work is generally desired by both individuals themselves and society, for reasons such as providing for oneself and others and being part of society [6, 7].

Research has shown that although people living with a severe mental illness have found meaning through engaging in various types of everyday occupations, employment was particularly important. Many meant that having a job was more important than being able to take care of oneself or having relationships [8]. Notwithstanding, people with mental health issues tend to have a broad perspective on what they see as “work”. It may not only include salaried work but also semi-market activities such as performing subcontracted work or providing different types of services at a day centre [9]. Some examples of activities seen as work are food catering, car washing, and running a cafeteria [10]. Even if these are not salaried employment they contribute to society and meet the needs of others as work does. We therefore use the term “productive activities” in the current study as a common denotation to cover salaried work and semi-market activities perceived as work.

It was shown in a study of young people with psychosis who attended day centres focusing on what they perceived as meaningful activities and whether they were longing for salaried work or not, that they were limited because of their mental health issues and thus did not think they had the capability for salaried work. However, they still wanted to do right thing for themselves and contribute to others, at the day centre as well as in the community in general. They also longed for a possibility to learn and perform new things, be able to go to school or a workplace, and they wanted to be economically compensated for their contributions [11].

Young adults with psychosis were also included in a study comparing work and related factors in three groups of people with mental illness [12, 13]. The two other groups were people who, in addition to their mental illness, had a substance use disorder or had newly arrived in Sweden as immigrants. Young adults with psychosis scored lower on work experiences and on resources for having a worker role in comparison to the two other groups, even though they were generally more satisfied than the others with their everyday activities and with their quality of life. This indicates that young people with psychosis both have specific needs in terms of resources and complicating circumstances. In-depth research is needed to shed further light on how this group envision their future possibilities for entering the labour or engaging in other productive activities, and what kind of support they would need for gaining employment.

The aim of the study was to explore how young adults with psychosis perceive their possibilities, wishes and support needs for gaining employment or engaging in other productive activities.

## 2 Materials and method

### 2.1 Design

The present study was part of a project named “Productive activities for people with mental illness: Supporting vulnerable groups”, focusing on people who were likely to have a special need for support regarding productive activities [12, 13]. The present part focused on young adults with psychosis and used a descriptive design with qualitative individual in-depth interviews as the method for data collection. The principles stated in the Declaration of Helsinki [14] were followed, and the study was approved by the Regional Ethical Review Board in Lund, Sweden (Reg Nr 2015/357). All participants gave their informed consent to participation.

### 2.2 Context

Psychosis units (*n* = 7) in two counties in the south of Sweden were invited to participate and six units consented. Staff in these units identified and selected patients who met the project’s inclusion criteria based on each unit’s register. The inclusion criteria were: a young adult, aged 18–30 years, and a main diagnosis of psychosis (F20–F29) according to ICD-10 [15]. The exclusion criteria were: dementia or intellectual disability, or current acute psychotic episode. A staff member at each of the units served as a key informant/gatekeeper [16] in the selection process, and contacted the identified young adults with psychosis and asked them about participating.

### 2.3 Participants

A total of 46 participants agreed to take part in the larger project and signed their informed consent. The present qualitative study was based on in-depth interviews with a purposeful subsample of these, representing variation with respect to age, gender, educational level, work experiences, and from each of the included six units. Eighteen participants were selected this way. Their socio-demographic characteristics are presented in Table 1. Most of the participants had completed high school. There were also participants who had started higher education, but none of them had managed to take an exam. Ten of the participants were occupied with productive activities at the time for the interview. Several of them had previously or at the time for the interview were engaged in short-term productive activities, such as internships or holiday work. There were participants who had occasionally had salaried work, and a few currently had salaried work they had received with the help of relatives. There were those who were in an organized occupation at a day centre, e.g., in dog day care service or a bakery project, and there were also a few who studied part-time or full-time with the goal of attaining a high school qualification. Although several of the participants lacked organized occupations at the time for the interviews, they occupied themselves by creating routines and organizing their everyday lives at home. All of them had ongoing contacts within the community psychiatry services, such as meeting a doctor or a nurse.

**Table 1:** Characteristics of the young adults with psychosis (*N* = 18)

Age; mean years (SD, range)	26 (2.8; 21–29)
Gender; number men/women/non-binary	11/6/1
Born in Sweden; number	15
Type of household; number
Single	11
Living with partner/family	7
Having children; number	2
Having a friend; number	18
Educational level; number
Completed 9-year school	6
Completed high school	12
Employment status; number
Employed	2
Student	4
Work rehabilitation	4
Unemployed	8

### 2.4 Data collection

The first author (ABG) and two project assistants, all occupational therapists, performed the interviews, which took place at the psychosis unit to which the participant belonged. The data collector first introduced herself and the intention with this study, and after receiving informed consent the participants were interviewed for about one hour each. The interviews, based on a semi-structured guide, focused on their everyday life, their experiences and hopes regarding productive activities, and their expressed needs for support to gain and maintain a job. Follow-up questions were used when adequate, such as “Can you please describe that further?” or “Can you give me an example?” The interviews were audio-taped and transcribed verbatim.

### 2.5 Data analysis

Data was analysed with qualitative content analysis based on the inductive approach proposed by Graneheim and Lundman [17]. Theoretically, content analysis can vary on a continuum, from manifest content that is close to the text, termed phenomenological description, to more latent content that is more distant to the text, termed hermeneutical interpretation. Additionally, the researcher can take a distant approach and conduct their analysis on an abstract level, or take an approach that is close to the text and keep the analysis concrete [18]. The current study was at the middle of the phenomenological— hermeneutical continuum, as was its approach on the abstract— concrete continuum. The trustworthiness of the analysis process was strengthened by the varied, professional and scientific backgrounds of the researchers (psychology and occupational therapy) and thereby also their preunderstandings. All of them, one man and two women, had experience from clinical work and research with the target group and of qualitative analysis. Data was read through several times in the first step. The text was then divided into meaning units and subsequently condensed into codes, which were sorted based on similarities and differences. Different sub-categories and categories emerged from the codes in a subsequent iterative process. The three authors then discussed and modified the sub-categories and categories. In order to further strengthen the trustworthiness [17] and to ensure that the essence of the participants’ experiences was captured, all authors reflected and discussed in the final step until consensus was reached.

## 3 Results

Four categories based on the experiences of the participants as vital for gaining and maintaining a productive activity were generated: “Wishing for a role in the community”, “Being my own coach”, “Needing personal support” and “Having a supportive workplace” (Table 2).

**Table 2:** Participants’ wishes and support needs for having work or other productive activities

Categories	Wishing for a role in the community	Being my own coach	Needing personal support	Having a supportive workplace
Sub-categories	Imagining myself in a working role	Having a personality that fits in	Having supportive relationships with family and friends	Coping with challenges at the work place
	Being one among others	Maintaining my motivation	Getting support from professionals	Expectations from employers, and how they could facilitate for work participation
	Access to training and education	Using my competence and abilities		Getting along with colleagues

### 3.1 Wishing for a role in the community

#### 3.1.1 Imagining myself in a working role

Most participants could imagine themselves in a student or working role. Those who saw themselves in a student role wanted to complete high school, go to college, and then work with something in line with what they were trained for. Participants had different preferences for how much they could be occupied with productive activities, ranging from 25 percent to 100 percent. They expressed hope and wished for a job in their future: “*There may perhaps be a possibility for me to get a job*” (P14), but they maintained that they had to recover and get healthier first. The participants commonly mentioned jobs in services and social care, i.e., they wished to work with children as a nurse or as a teacher, or with elderly people. One participant spoke of becoming “*a volunteer* ⋯   *for example, they suggested at the Employment Service that could become a volunteer for pensioners and keep them company, I’m used to it, and it would be fun*” (P9). There were also those who wanted to work as case managers or work as shop assistants. Some of them also dreamt of blue-collar work as, e.g., a janitor within the municipality. Some gave examples of situations where others had suggested what they should do, such as when a career guidance counsellor suggested to a participant to train in the nursing profession.

#### 3.1.2 Being one among others

The participants wanted to be one among equals, feeling accepted and socializing with peers, not only with their families, who were the ones they met most often. Participants experienced it as difficult to get in touch with others, because they had lived withdrawn lives due to their mental health issues. Even though it would take courage to leave safe environments as the mental health care and day centres, they longed for being part of a group, and meeting people without any mental health issues.

There were participants whose goal was to get a qualification, because a professional title was an important aspect of being one among others. That would also lead to work, development, and a possibility to make a career, which in turn would improve their well-being and a sense of being proud of themselves: “*if I get a title and do what is right for me then I think I*’*ll feel well*” (P13).

Gaining an employment or other productive activity was seen as a means to enter a social context, although it had to be on their own terms. Meeting other people who were in the same situation as themselves was also positive. If they felt accepted and respected, the participants experienced real companionship and being one among others: “*Yes, but it was the atmosphere, everybody was pleasant and talked to me, like, and I was able to do it at my own pace, that was what had been the best*” (P10). Moreover, having a job also involved longing for a partner and friends.

Furthermore, having their own housing and earning money would mean that participants could adapt to the norm, become financially independent and be able to pay off their debts. Participants would like to live a tolerable life, and for example be able to go to a store and buy what they needed without worrying about whether they could afford it or not:

*I have dreams of being able to work and get by financially, that’s a dream I have, to be financially independent. And then that it also means a lot to me /* ⋯  */ it means that in one’s own eyes and in the eyes of others to maintain some... decency and respect /* ⋯  */ and then on top of that you then sort of maintain a certain self-respect and respect for.... or others respect you and /* ⋯  */ and to assert myself* (P13).

#### 3.1.3 Access to training and education

Gaining employment or engaging in other productive activities was not seen as an easy task. There were participants whose goal was to first get a qualification, while others were more focused on getting a job directly. The participants said that they needed help to become aware of their mental and physical condition as well as their limitations before entering that job. They also needed training to become independent and to develop their competence. Some of them wanted an opportunity to try and test their skills under the guidance of staff and at their own pace at a day centre first. The first thing might be to do a little when they were inspired, and then taking part in internship or vocational training. Others wanted support from the social insurance office and the employment agency, i.e., the mainstream interventions directed towards getting work. There were those who expressed a need for help to get on with studies or work, while others did not wait for any help and arranged an internship themselves. Some participants sought a safe haven for testing a job or other productive activity, while others wanted to go into working life directly: “*I think it would have been fun to be able to advance and eventually, because.... a proper job is more challenging, I think*” (P4).

### 3.2 Being my own coach

#### 3.2.1 Having a personality that fits in

Participants described various characteristics of their personality, which they meant could facilitate or limit their possibilities to gain employment, or to be engaged in productive activities. There were those who meant that their strengths, such as being positive and happy, and good at socializing, would be facilitating. Being an optimist and looking at the bright side of life will make it easier to connect with others, colleagues as well as employers.

Furthermore, it was experienced as important to be an honest person, an individual who others can trust. This could concern being open-minded and able to talk about things related to work, but also being on time for work: “*I have a voice inside me that says that I have to do a good job on the internship* – *I have to get up and out of bed* – *that voice is like a support for me*” (P13).

The participants described personal limitations, such as having difficulty to plan and develop a good structure in everyday life. Another limitation was being sensitive to stress, which could concern doing different things simultaneously or be related to financial difficulties. The difficulty in dealing with stress could generate a feeling that something was wrong with them. It was difficult for them to perform work and other productive activities when they were highly stressed. Some of the participants conveyed that they could suddenly become tired and unable to do their chores, or have difficulties staying focused:

*I disappear in my own world and when I then be*  ⋯  *. when I become like that in those situations*  ⋯  *. I become tired or am unable to focus properly and then it would be good if they could accept, that they could accept it and think, yes, go and sit down and have a rest* (P5).

#### 3.2.2 Maintaining my motivation

Participants spoke of needing to maintain their motivation in situations when it could be difficult to overcome resistance and changes in everyday life. Being motivated could concern a desire to continue being alive or wanting to be part of a group and not feeling lonely. Making one’s own decisions was not easy for the participants, but they would often manage by triggering their motivation:

*As long as it*’*s the right thing then I want to be part of it, but if it feels as though nothing is happening*  ⋯   *then it*’*s not so good, then I*’*m prepared to have to press on, that I*’*m the one who has to press on* (P18).

There were those who felt that difficulties concerning their motivation at home affected their ability to perform occupations outside the home, e.g., to cope with a job. Some talked of a lack of interests, while others meant that they were not good at getting up in the mornings, and others stayed in bed all day. Some who lived alone were not always motivated to take care of their hygiene. Staying at home was perceived as boring and monotonous, and one of the participants said:

*I don’t like cleaning and I don’t like eating and I don’t like cooking and I don’t like washing, so I don’t do that much.*  ⋯   *I have to do it and I kind of do sometimes, but most of the time it doesn*’*t get done. I....so the thing is that I.... well, on one level I have such a hard time seeing the point with cleaning, because it always gets dirty again and you know, I found everyday life difficult /* ⋯  */ due to it just gloomily plodding along /* ⋯  */ I always want something to happen, so*  ⋯  *.the whole idea of everyday routines become* ⋯  *it becomes like a prison for me and I can’t handle it* (P15).

Just being at home all day, with no routines and structure, was perceived as unsatisfactory, when they just watched the television or simply did nothing after waking up, making breakfast, and cleaning up. The participants spoke of having difficulty in planning ahead, getting away from home and managing a job: “*have been too tired or too lethargic and I haven*’*t been able to keep going a whole day and work*” (P16).

#### 3.2.3 Using my competence and abilities

Participants described the competences and abilities they had, which could be useful when being at work or engaged in other productive activities. There were those who thought good grades in school, e.g., in mathematics, were useful. There were also participants who wanted to use their education to help others, e.g., someone who had a nursing education. Other participants said that they were good with children and felt they had the ability to work with them, e.g., as a teacher. In general, the pursuit of an interest was a good way to use their abilities, e.g., one participant who had a voluntary job: “*got a job at an ecological farm for children, because I*’*m interested in ecological things and I*’*m interested in children*” (P6).

There were participants who experienced that they were good at practical/physical tasks but were never good at precision and completing details. Others said that they had previously found it difficult to concentrate on tasks, but that they had improved now and were able to be focused when carrying out a task. There would be possibilities to feel well when they felt they were in balance and had structure in everyday life. The participants conveyed that one way to strengthen their mental well-being and develop their abilities might be physical training. Others had tested going to the swimming baths or learning foreign languages to boost their skills, although that had not worked for them. Having a mental illness may also entail difficulties in relating to others, such as making and keeping friends, which in turn could have an impact on their ability to manage jobs and other productive activities, even though they were highly motivated for this: “*to be able to cope with the symptoms and feel that I am in control at work, I*’*d like to be able to do all the aspects of the work*” (P10).

### 3.3 Needing personal support

#### 3.3.1 Having supportive relationships with family and friends

The participants spent a considerable amount of time at home alone, but they also had a social network of family and friends, who knew how mental health issues could affect the individual in everyday life. That type of supportive relationship was perceived as a prerequisite for being able to get to studies, a job, or other productive activities, e.g., a wake-up call in the morning. Parents would support with practical things, such as transport to and from a job or other productive activity, sometimes as a daily routine. Parents also encouraged the participants to relax and be calm and wanted to help them in their recovery process. Both families and friends were supportive when it came to looking for a job, e.g., how to write a CV, or just answering questions about how to get a job. Sometimes parents and other relatives had contacts in working life; in fact, those participants who had a job, were employed in their parents’ company or workplace. One participant, who did not get the job that he/she had applied for, spoke about support from friends: “*It was of course difficult, but I talked with a few friends then and it happens to everyone, then it felt better when I could think of that. It can happen to anyone*” (P1).

#### 3.3.2 Getting support from professionals

Some of the participants already had support from in-house staff from the social services, others expressed a need for such support. They needed help to carry out household chores, to continue medication, and to get to their productive activity. Another kind of support that was spoken of was to have an emphatic and active listener, with knowledge of everything that needs to be considered before looking for a job or other productive activity. It seemed important to have all the time one needed to recover, and not speed up the process, everyone should take their time at their own pace.

The participants were generally satisfied with the support they received from the mental health care services, even though they sometimes thought that the staff ought to help them even more and alleviate their relatives’ situation. The participants expressed that they had not only received supportive therapy and medication but also support concerning how to manage one’s finances and finding motivation for studying and managing one’s job. This type of support was seen as valuable;

*I talk to her, the nurse, so we have... try to have a conversation once a month. And then I’ve said that it feels good to have someone to exchange ideas with like that, because it’s a little... it feels good, it usually helps a little, I think so. So that you don’t... sit at home by yourself like that, because it can get a bit isolated* (P4).

The participants emphasized the importance of having connections with people who could pep them up. It could be staff from the social insurance office, the employment agency, and/or Supported Employment programs. The participants who had received support from case managers perceived them as companions in the community, who could help with one’s personal economy, but also with compiling CVs and personal letters that participants submitted to potential employers. They had received thorough guidance from their case managers regarding how to manage everyday life prior to starting an internship or a job. Some participants had also received wake-up calls so they could be at their job on time. The participants found it important to have case managers who listened to them and could take their fluctuating mental health into consideration. A case manager needed to be calm and let participants do things at their own pace, i.e., someone “*who listens and tries to adapt what one does to how much one is able to do*” (P8). The participants found it desirable to have case managers who were close, but still not intrusive, someone who gave them constructive feedback at different stages and at the right time.

The participants expressed that staff whose job it was to assist them should see to the individual and their characteristics in order to be able to help them to an adequate job or other productive activity. They could, however, give several examples of the opposite and of feeling confused in the contact with the authorities. They said that the social insurance office and the employment agency generally had long processing times, and that different authorities referred to each other and no one took responsibility for the whole. They were given numerous forms to fill in, for example, to receive financial support, but the participants had difficulty understanding how the system works and which rights they have. For example, there could be difficulties when they wanted to study a single course; and they hesitated about studying, because they did not know the rules for sickness benefits versus study loans in the Swedish system, “*when I*’*m unable to study full-time then it becomes very wrong*” (P5). There could also be difficulties in terms of the professionals’ attitude towards the participants, e.g., when they went to college and met teachers who did not understand that students may have specific needs.

### 3.4 Having a supportive workplace

#### 3.4.1 Coping with challenges at the workplace

A few aspects facilitated participation in productive activities, these included the alignment of the work tasks with their personal interest, the challenges being just right for their capabilities, and the close vicinity of the workplace to their home. They needed calm jobs that did not stress them. Working with productive activities other than salaried work was positive as the participants could choose what they wanted to do and for how long. This often entailed a slower pace and adjusting the work tasks to their capacity. There were participants who enjoyed more monotonous routine tasks, while others needed greater variety to be motivated to continue working. Being allowed to stay at a workplace for a longer time, so that the participants could learn the work and develop new skills was an advantage: “*you know and they are very good at coaxing my strengths, they are very good at and you know getting me involved in what they*’*re doing*” (P15).

It could be a challenge to cope with a job or other productive activity. The participants struggled with concentration, and it was more difficult if they found their work tasks less interesting:

*Maybe it was because it didn’t work very well, I didn’t get very well /* ⋯  */ I can*’*t manage those conveyor belt jobs. No, it doesn’t work for me. I need to have some variety. It was like in industry, it’s a bit more relaxed there, you can’t lie about that, but like the three jobs I enjoyed, they were self-paced jobs, free jobs. You went and sort of took care of what you needed....it wasn’t so much of a routine, that it was the same thing every day or so* (P2).

A lack of concentration made it difficult to understand things, especially if theoretical matters were concerned, and they needed to ask many questions and receive detailed instructions. There were those who felt that the work tasks were too difficult due to their mental health issues. Other circumstances that were inhibiting were if the work was solitary or had to be done under time pressure, or if the participants felt forced – that society expected them to have a job or they felt demands to extend their working hours with the consequence of being completely exhausted by the weekend. The participants also talked of an uncertainty concerning their prospects of getting a job. They also said that having responsibilities might be a burden:

*It’s a bit scary with the project [work experience at a garden centre], although I think it’s a lot of fun and so, I hope it continues... and at the same time I don’t know how much I can do, because I’ve been that bad so I hope there*’*ll be other people there who can take on more responsibility* (P6).

Meeting other people who were in the same situation as themselves was also positive. Other job benefits were having something to do, having routines and regular times, and being able to perform various work tasks. Working in a café could be fun and instructive, as could working in a daycare service for dogs or painting and refurbishing furniture, or providing various types of assistance at nursery schools and schools.

Although the participants were mostly positive about being involved in productive activities other than paid jobs, there were those who were negative about providing “free services” for which they received no more than a lunch as compensation.

#### 3.4.2 Expectations from employers, and how they could facilitate for work participation

The participants spoke about there being only a few employers who want to hire someone who is on sick leave due to mental illness. The participants often lacked higher qualifications and thus had to apply for low demand jobs. They had sometimes been able to get a job or other productive activities by using that strategy. The participants had mixed experiences regarding the expectations of and the encounters with the employers. One employer could expect the person just to relax and do nothing, and the participants then had had to talk about themselves and how they had got on at school:

*I got the feeling that they are very much used to the fact that those who come there don’t know what they want, that they may not be ready to do anything, that they just relax, and then it became a bit like they I thought I was the same and it’s a bit annoying, but I didn’t want to just scream out loud, but I just had to sort of explain my thoughts and wishes and dreams and sort of tell them how I’ve been feeling a little and so, so I guess they have begun to understand that I wasn’t the type to skip school or anything like that, so for example when it comes to school then, I was one of those who behaved well*” (P17).

The participants had sometimes met employers who organized an internship or employment without waiting for decisions from the authorities. There were employers who were supposed to act as mentors for the participants but failed to do so. Others paid attention to what the participants did well and encouraged them. An employer could function as a role model by, for example, being considerate and generally educated and calming the person down in stressful situations.

It was extremely important to have an understanding mentor who had long professional experience and good knowledge about what was to be done and could also explain how. The mentor could be the employer or some experienced employee. They functioned as good mentors and could adjust the tasks to the individual, so that there was neither too little nor too much responsibility. Such mentors also wanted to get to know the participants, keep in touch and were someone the participants could talk to about their mental health issues, and could provide the needed support. All of this made it easier for the participants to enjoy work, and to feel safe.

#### 3.4.3 Getting along with colleagues

To become a member of a group of colleagues at a workplace and socialize with people the participants had not previously known could be intimidating. It could also be difficult to deal with a tough jargon at work. Some thought that it might help if the colleagues knew what type of mental health issues they had. They had experiences from work colleagues who facilitated for them to get the work done because they knew about their difficulties, such as concentration problems. Others maintained the opposite, that it would not be necessary to know everyone’s secrets. Some spoke of experiences of prejudice, e.g., colleagues joking about people with mental illness;

*I had a fear or what can I say, that I could be ostracized.... if I had, for example, gone to a shop [that had a certain reputation], and if someone said that...then I might have been seen as being somewhat odd and different.... I don’t know, that’s my dread* (P10).

There were also experiences of being spoken to as a child, or hardly being treated as a human being at all. In some cases, and to some extent, this could depend on the participants themselves: “*I didn*’*t feel well myself, so I don*’*t think that they could have done more, that could have helped me, because I was so suspicious about them all, so even if they said something positive I interpreted it in a totally different way*” (P16). The positive experiences that were mentioned concerned being greeted with a friendly attitude at the workplace, having lunch together, talking and joking with each other, and sharing interests with others. They could then also share things that were not workplace-related, such as wanting to get a driver’s license. It was easier for the participants to be more open about themselves with open-minded colleagues.

## 4 Discussion

The findings showed that the young adults with psychosis who participated in the current study desired a worker role in the community. They needed to be their own coaches, but they also needed support from others in their environment, including supportive workplaces when attempting to reach this goal. Although all of them wanted something more “than just being at home” and doing nothing, the participants’ wishes varied; some of them imagined themselves as students or employees while others wanted some type of semi-market productive activity. This shows that they are not a homogeneous group, but individuals with different needs.

Paid work is desired by people with mental health problems, for reasons such as providing for oneself and others and belonging to a social context [6, 7]. The participants in this study conveyed that having an employment meant being one among others, including having beliefs in a future worker role. They longed to fit in and be in a context, socialize and earn their own money. The identified importance of being in a context may be linked to Hamell’s [19] concept of belonging, which in turn can facilitate for the individual to become the person he/she wants to be. The current findings are similar to those in a study by Gunnarsson and Eklund [11], showing that young adults with psychosis who attended day centres desired a future worker role, even though they had never had such a role. The findings of optimism about the future in that study and the current one contrast with those in a study by Jansson et al. [20] on work prospects among people with substance use disorders. These had previously had a job, but they now had only low expectations regarding fitting in and getting a new job, which they attributed to previous negative work experiences, including stigma. Helping people with mental health problems maintain their optimistic views on future work prospects that have been found among young people would be an important task for the support systems involved, including the healthcare and social services, employment agencies, and workplaces, and would require targeted education for the staff groups involved. The findings from the current study regarding support needs and measures could be included in such educational efforts.

It is important to note that paid work or some other type of productive activity was not only desired by the individuals themselves in this study, but they also conveyed that it was desired by society. This is in agreement with Nilsson [21] who maintained that exclusion has a high price, partly in terms of direct costs, such as assessments, visits to healthcare services, and the economic support individuals need for their livelihood. In addition, there are substantial indirect costs in terms of production losses for the young unemployed adult, but also for close relatives who may need to accompany their relative to, for example, doctor’s appointments, or even be on sick leave themselves. A complicating factor for the participants in this study was employers who were not available or who put the same demands on an individual with mental health issues as on others at the working place. To facilitate for people with mental health problems to be part of working life, there needs to be a natural flexibility so that the work demands can align with the mental health state of the person, which may vary from one day or period to another. This is obvious from the current findings and has also been shown in previous research [22]. This may allow for small steps to be taken, and each of these would be important for both the individual and society and bring about positive economic consequences. For example, being able to stay at a workplace or as a trainee even on a bad day can diminish the need for mental healthcare or alleviate the burden otherwise placed on a close relative.

The participants in this study said that having a personality that fits in, e.g., being motivated and having a positive mindset, makes it easier to get a job, while withdrawal and cognitive impairments make it more difficult to maintain work participation. This is in agreement with Lindhardt et al. [23], who showed an association between withdrawing, unemployment and being young with psychosis, and with Harvey et al. [24], who found that social competence and cognitive impairments are associated with fewer possibilities for employment, and also with Brouwers [25], who stated that social stigma implies a high risk for unemployment. According to the current findings, expectations of having a positive mindset, and at the same time having various impairments, could be experienced as stressful, which accentuated the difficulties in entering the labor market. However, the role of cognitive impairments should not be overrated. Caruana et al. [26], when investigating the relationship between cognitive skills, job complexity (higher or lower cognitive demands) and managing to maintain an employment over time, argued that what mattered was that the individual was motivated for the job, enjoyed it, and that the job suited their personality.

To reach the goal of being engaged in work or other productive activity, the participants needed a supportive encounter from different sectors in the community: their families and friends, healthcare professionals and various authorities, but also from present and future employers and work colleagues. A crucial aspect of experiencing such support was a mutual and trustful relationship, which the participants in this study felt when they were seen and confirmed as the individuals they were. Unfortunately, however, the participants in this study experienced difficulties in their communication with the authorities, which could be seen as a barrier when most of them were in the initial step of the process of job seeking. The relationship between client and helper is seen as essential and as the core of the support in virtually every sphere of the healthcare and social services [27], which is also true in the Supported Employment (SE) context where positive staff attitudes were essential for successful implementation [28]. It is vital that the support is based on facilitating for the person with mental health problems to find hope, restore identity, find meaning in life, and take responsibility for recovery [29]. Hamovitch et al. maintained that a good therapeutic relationship is closely linked to person-centered support, which could result in good outcomes [30]. A crucial aspect is that the individual feels respected and involved in their own care and has an open communication with their supporter, who they experience as having both personal and professional expertise [31]. A person-centered approach can encourage more of a holistic and health-oriented focus and less of one on symptoms and mental illness [32]. Such an approach would be useful for professionals, as well as future employers and colleagues at workplaces, and supports a focus on the individual’s strengths, i.e., interests, talents and skills, in the working relationship [33].

Only one of the participants spoke about being involved in SE. We do not know whether the participants had been offered, or tried SE previously, but participation in SE could have a positive impact on the incidence of participating in productive activities [34]. Those authors showed that participation in programs such as SE and Early Intervention (EI) had a positive impact for young adults with psychosis in obtaining an employment, but to maintain the employment remained a challenge. Continued support, when the individual’s needs and wishes are listened to, is therefore needed from relatives, professionals, and presumptive employers and colleagues who aim to provide support for the individual when seeking, obtaining and maintaining an employment.

The participants also had difficulties in their communication with employers, whether they should tell them about having a mental illness or not, and whether it would benefit them in terms of completing tasks and contacts with colleagues. Severe mental illness has been found to be a significant barrier to employment in previous studies [35, 36]. However, the participants in this study hardly mentioned this as a barrier or stigma, possibly due to them still being far away from an employment situation and had therefore hardly ever experiencing it.

Although paid work is generally desired by both society and the individual for reasons such as providing for oneself and others and being one among others, the findings from this study indicated that the participants were far from having paid work. They expressed hopes of being able to work as, for example, a teacher or a nurse, but the reality right now was that their first priority was to get structure in everyday life and hopefully get a semi-market position. On the other hand, as described by Boardman et al. [37], the fact that the participants were occupied with their daily routines might reflect that professionals, as well as staff at workplaces, are focused on the individual’s psychological status, and not their capacities and skills. This study can thus be useful as an eye-opener, initiating a process that, in line with statements from WHO [38] as well as Killeen [33], focuses on the views, priorities and resources of young adults with psychosis, and not mainly on their limitations.

Besides SE, which implies direct training with support in a real workplace [28], other measures to buttress job establishment among people with mental illness have been proposed as well, such as increased work readiness [39]. The latter study concluded that focusing on individuals’ views of their worker role, especially their expectations on job success, would be important in all efforts to support towards work or other productive activities. The implications from the current study are similar, that a broad approach is needed, including both direct paths to work and possibilities for developing a self-image as a working person, which could be included in any type of social and healthcare services.

The trustworthiness [17] of this study needs to be taken into consideration. Using a descriptive design with qualitative in-depth interviews to present how young adults with psychosis experienced their wishes and needs for getting and having a productive activity strengthened the credibility. Moreover, the credibility was strengthened when we recruited participants who represented variations in terms of age, gender, educational level, work experiences, as well as all of the six units included in the larger project. However, we did not manage to recruit any participant younger than 21 years, maybe because at that age they had not yet attempted to enter any productive activity. Furthermore, the participants contributed with a variation of experiences from getting and having a productive activity, which further strengthened the credibility. However, variation may sometimes be a limitation, for example, in terms of transferability. Showing what reality looks like is a strength; people with psychosis are not a homogenous group.

The dependability was strengthened by the thorough description of the analysis process. The fact that the first author interviewed some of the participants, and had an inside perspective, while the other authors had an outside perspective, together with the authors’ varied pre-understandings, strengthened the dependability, further supported by the fact that the analysis was discussed until consensus was reached. Finally, even though a descriptive design with a qualitative approach is based on a small number of participants, and the current study was conducted in a Swedish context, we argue that the variation obtained concerning the experiences of the wishes and needs related to getting and having a productive activity also renders some transferability to the study, although limited to similar groups and contexts. Another weakness could be the absence of interviews of caregivers and employers. A triangulation of findings could have strengthened the transferability. Moreover, the number of presumptive participants who declined to be interviewed could not be calculated due to the selection procedure relying on gate-keepers. It is possible that the more severely ill people were among those who declined.

## 5 Conclusions

This study showed that young adults with psychosis were far away from having paid work, but they desired to have a worker role in the community. They needed to be their own coaches, but they also needed support from others in their environment and wanted supportive workplaces while attempting to reach their goal. The findings also showed that the young adults had different experiences of what they needed, indicating that they are a heterogeneous group, i.e., individuals with different wishes, needs, abilities and skills. It is thus of importance that professionals, employers, and policy-makers have a person-centered and holistic approach, listening to the individual themselves and adapting tasks and workplaces to match their needs. They should also acknowledge the importance of both directs paths to work, via employment offices and SE, and measures that can be taken in any type of services to help the individual keep a self-image of being capable of work and believing in having a future worker role.

## Ethical approval

The study was approved by the Regional Ethical Review Board in Lund, Sweden (Reg. Nr. 2015/357).

## Informed consent

All participants received oral and written information about the study and provided written informed consent. All procedures were in accordance with the ethical standards of the responsible committee on human experimentation and with 1975 Declaration of Helsinki, as revised in 1983 and 2004.

## Conflict of interest

The authors declare that they have no conflict of interest.

## Data Availability

The datasets generated and analysed during the present study are not publicly available due to ethical considerations but could be made available from the corresponding author on reasonable request.
